# A nationwide study exploring the factors associated with psychological resilience during the COVID-19 pandemic in Singapore

**DOI:** 10.3389/fpubh.2025.1516829

**Published:** 2025-04-24

**Authors:** Savita Gunasekaran, Eng Hong Tay, Saleha Shafie, Shazana Shahwan, Peizhi Wang, YunJue Zhang, Pratika Satghare, Sing Chik Tan, Michael Y. Ni, Phyllis Lun, Siow Ann Chong, Mythily Subramaniam

**Affiliations:** ^1^Research Division, Institute of Mental Health, Singapore, Singapore; ^2^School of Public Health, Li Ka Shing Faculty of Medicine, The University of Hong Kong, Pokfulam, Hong Kong SAR, China; ^3^Saw Swee Hock School of Public Health, National University of Singapore, Singapore, Singapore

**Keywords:** COVID-19, pandemic, resilience, public mental health, Singapore, Asia

## Abstract

**Background:**

The COVID-19 pandemic is a global adverse event that affected many individuals’ well-being. Resilience is an essential component that allows one to cope during stressful events such as the pandemic. Not many studies have longitudinally explored changes in resilience across time during the pandemic in the Southeast Asia region. The current article investigates resilience and the sociodemographic and psychological factors associated with resilience across two waves of survey of a Singapore adult population.

**Methods:**

The study was conducted across two timepoints from May 2020 to June 2021 (T1) and October 2021 to September 2022 (T2). 1129 participants partook during T1 (response rate = 54.8%) and 858 participants partook during T2 (response rate = 76.0%). The questionnaire included sociodemographic information and measures such as the Brief Resilience Scale, Patient Health Questionnaire-9, Generalized Anxiety Disorder Scale-7, the stress component of the Depression, Anxiety and Stress Scale, and four COVID-19-related stressors. Generalized estimating equations (GEE) were utilized to investigate the relationships adjusting for timepoints.

**Results:**

Most participants had normal levels of resilience (*M* = 3.61, SD = 0.62), and resilience scores did not differ much over time (*p* = 0.852). Males, younger adults, university-educated, employed individuals, and individuals living in private housing had higher levels of resilience. Higher levels of anxiety symptoms, depressive symptoms, stress, and one specific COVID-19-related stressor (i.e., employment concerns) were associated with lower levels of resilience. Individuals who reported having moderate to severe depression and anxiety symptoms were more likely to have low resilience.

**Discussion:**

The findings suggest that resilience levels remained within the normal range and unchanged over time, reflective of the population’s ability to cope with the pandemic. However, there is still a need for more targeted interventions for individuals who are more vulnerable to lower resilience. Continued research is also needed to understand the long-term psychological effects of the pandemic.

## Introduction

The global outbreak of the novel coronavirus disease (COVID-19) was described by the World Health Organization as a public health emergency in January 2020 (1). On top of the threat to physical health, many lives had been upended by the strict pandemic measures, such as lockdowns and social distancing measures (2, 3). The widespread contagion of the coronavirus alongside pandemic measures led to income loss, loneliness, distress, and social isolation for many (4, 5). As such, there was considerable concern about the psychological consequences of the COVID-19 pandemic (6). It was evident that there was a strong need for mental health support during these times, and protective factors such as resilience could have played a key role in sustaining mental health (2, 7, 8). Various definitions of resilience have been proposed throughout the years (9). In this study, we conceptualized resilience as the ability to bounce back or recover from adversity (10). From this perspective, resilience is viewed as a dynamic process understood through patterns of responses to challenging situations or events (11). It can vary within one individual across time and circumstances and thus can be captured using within-individual response trajectories across time (11, 12). The COVID-19 pandemic presents an opportunity to explore resilience during a prominent global adverse event (13). Having insight into resilience is essential in understanding the healthy adaptation to stressors, which would be beneficial in implementation efforts to aid individuals in coping with adversities (14).

To date, a considerable number of studies have sought to explore resilience during the COVID-19 pandemic and the common factors associated with it. Some of these studies have identified that females have diminished levels of resilience (Brief Resilience Scale; BRS scores) compared to their male counterparts (15–17). During the pandemic, the dire need for interventions targeted at boosting resilience for individuals with lower socio-economic status was highlighted, given their susceptibility to stress-related psychological symptoms (18). In a cross-sectional study conducted by Riehm et al. (17) in the United States, it was revealed that adults living below the federal poverty line were more likely to have lower BRS scores as compared to adults above the poverty line. The study also revealed that adults with a graduate degree had higher odds of high resilience as compared to their counterparts with a high school education or below. Besides socioeconomic status, a longitudinal study demonstrated that individuals aged 18 to 34 had lower resilience scores on the BRS, indicating that young adults are more vulnerable to poorer resilience (19). Other studies utilizing different scales have also demonstrated that resilience and proactive coping were higher among older adults during the pandemic (20–22). Additionally, while the relationship between marital status and resilience has not been largely explored during the pandemic, some studies have suggested that married participants experience heightened anxiety levels as compared to their unmarried counterparts, and that widowed/divorced individuals experience worse anxiety as compared to their married and single counterparts (23, 24). A cross-sectional Turkish study utilizing an adapted BRS revealed that resilience did not differ significantly across marital status, but more research is needed for conclusive associations (25).

Additionally, symptoms of anxiety, depression and self-reported stress were the most prevalent psychological reactions to the pandemic (26). This is also observed in a systematic review that reported a high prevalence of symptoms of anxiety, depression and stress across multiple countries (27). Nationwide studies such as the one conducted by Dragan et al. (28) in Poland utilizing the Patient Health Questionnaire-9 and Generalized Anxiety Disorder-7 scales reported that about 25.7 and 43.9% of the sample reported having moderate to severe levels of depressive and generalized anxiety symptoms, respectively. Additionally, a longitudinal study investigating the trajectory of anxiety during the pandemic revealed that generalized anxiety was associated with an increased risk of somatic symptoms (29). Given their prevalence and risk during the pandemic, there is importance in addressing these specific psychological distresses. The pandemic is associated with numerous stressors that might affect individual resilience. The most common stressors identified included worries about family members contracting COVID-19, infecting someone else without knowing, and financial issues following COVID-19 (13). The relationship between psychological distress and resilience has been well documented during the pandemic – a longitudinal study conducted in Australia revealed that normal levels of depression, anxiety, and stress were associated with higher scores on the BRS (30). Another two-wave longitudinal study conducted in China revealed that resilience among adolescents, measured using the Chinese Positive Youth Development Scale, was negatively associated with depression and anxiety six months later (31). Additionally, Barzilay et al. (13) also demonstrated that generalized anxiety symptoms, depressive symptoms, and concerns about COVID-19-related stressors were linked to lower BRS scores. Another study conducted in Spain identified that depressive symptoms were a predictor of poor BRS scores in clinical populations, but no associations were found for healthy controls (6).

Several studies have investigated resilience during the COVID-19 pandemic, including some longitudinal research examining the period following the outbreak (32, 33). This is particularly important, as existing literature highlights the persistence of psychological distress long after the pandemic has ended (34). However, a look into existing literature suggests a scarcity of studies examining resilience post-lockdown in Southeast Asia. There is a need for more studies in the region, considering evidence of cross-cultural differences in resilience among different countries (35). There is value in understanding the mental health trajectory in different populations as it can contribute to the informing of nationwide policies, help to determine the impact of pandemic measures on population mental well-being, and plan out the allocation of resources. Therefore, the present study looked at changes in psychological resilience in Singapore across two waves during the pandemic spanning early 2020 to late 2022.

Singapore is a Southeast Asian country with a population of approximately five million individuals. The majority of the population is ethnically Chinese (74.3%), followed by Malay (13.5%), Indian (9%) and Others (3.2%) (36). As of October 2022, there were 1.9 million reported infections and 1,620 deaths in the country due to COVID-19 (37). Singapore went through a nationwide partial lockdown from 7th April 2020 to 4th June 2020, followed by a planned reopening. Restrictions due to the Delta and Omicron Variant started on 8th May 2021 and ended on 29th March 2022. COVID-19 restrictions were removed by 29th August 2022, except for mandatory mask-wearing in public transportation and medical settings like hospitals and clinics. The relative and inevitable uncertainty regarding the COVID-19 spread, alongside the many other consequences of the pandemic, could have been a source of psychological distress among many Singapore residents. An understanding of psychological resilience among the Singapore population would be beneficial in the preventive efforts not only for the current pandemic but for future pandemics and other events of a similar nature.

The present study utilizes an exploratory approach to understand the changes in individual resilience during the pandemic within the general population of Singapore across two waves spanning early to mid-pandemic. As we viewed resilience as a dynamic concept, we hypothesized that individual resilience levels would decline across time due to the multifaceted stressors introduced during the course of the pandemic. Secondly, we aimed to investigate the association between sociodemographic variables and perceived depression, anxiety, stress, COVID-19-related stressors, and resilience using data from both waves. We also aimed to further investigate the relationship between resilience levels and severity of depressive and generalized anxiety symptoms.

## Methods

### Sample

The present study was part of a larger study investigating well-being and resilience during the COVID-19 pandemic in Singapore. Ethical approval for the study was provided by the National Healthcare Group Domain Specific Review Board. Institutional Review Board protocol number: 2020/00462 and 2021/00566. A total of 1129 participants took part in the first wave of the study from May 2020 to June 2021. Participants were individuals from the general population who participated in the Singapore Mental Health Study in 2016 and had provided consent for re-contact. 50.9% of the participants were female, and 49.1% of the participants were male. The mean age was 46.70 (SD = 16.45). Most participants were Chinese (76.1%), university or pre-university educated (33.3 and 28.3% respectively), married (62.5%), and employed (71.5%). Table 1 depicts the summary statistics of the sociodemographic data of the participants at the first wave of the study. Additionally, a detailed methodology has been described in an earlier article (38). The inclusion criteria were (1) Singapore citizen or Permanent Resident (PR), (2) aged 21 years and above, (3) ability to speak in English, Bahasa Melayu or Mandarin, and (4) available for an interview via ZOOM video conferencing platform or face-to-face. The exclusion criteria included (1) severe physical or mental disorders that limited participation in the study and (2) not staying in Singapore during the survey period. Out of these 1129 participants, 858 participants took part in the second wave of the study conducted from October 2021 to September 2022. The response rate for the first timepoint and follow-up was 54.8% (after excluding those whose contact details were invalid) and 76.0%, respectively.

**Table 1:** Summary statistics of sociodemographic variables in Timepoint 1 (*n* = 1,129).

	Weighted percentage (%)	Unweighted frequency
Age groups		
21–34	27.5	426
35–49	26.9	361
50–64	26.1	219
65+	19.5	123
Gender		
Female	50.9	527
Male	49.1	602
Ethnicity		
Chinese	76.1	398
Malay	13.0	278
Indian	9.0	324
Others	3.3	129
Highest education attained		
Below primary	13.8	51
Secondary school	24.6	151
Pre-university^1^	28.3	398
University	33.3	529
Others		
Current employment status^#^		
Unemployed	6.9	235
Economically inactive	21.6	570
Employed/Self-employed	71.5	309
Marital status		
Never married	27.4	363
Married/Cohabiting	62.5	681
Divorced/Widowed/Separated	10.2	85
Do you have any children?		
Yes	62.3	647
No	37.7	482
Monthly personal income (SGD)^#^		
Below 2,000	36.2	309
2,000 to 3,999	28.9	342
4,000 to 5,999	15.6	228
6,000 to 9,999	11.0	142
10,000 and above	6.3	93
Housing type		
HDB^2^ 1/2/3 room	16.1	163
HDB^2^ 4/5 room/Executive/Jumbo	67.5	747
Private housing	16.4	217
Brief Resilience Scale*		
Low	13.3	143
Normal	74.5	846
High	12.2	140

^#^Personal income and employment status, *n* = 15 missing data. Housing type, *n* = 2 missing data. *Cut-off scores for BRS: low (0–2.99), normal (3.00–4.30), high (4.31–5.00). ^1^Pre-university includes Junior College, Vocational Institutes, Institute of Technical Education, and any form of Diploma. ^2^Housing Development Board (HDB) refers to public housing in Singapore. As of 2021, about 80% of Singapore’s population live in houses developed by HDB.

### Materials

#### Sociodemographic variables

Age, gender, ethnicity, highest education level completed, employment status, monthly income, marital status, having children, and type of housing were collected as part of a structured questionnaire. Employment status was collapsed into a three-level categorical variable (i.e., economically inactive, employed, and unemployed). Marital status was collapsed into a three-level categorical variable (i.e., never married, married/cohabiting, and divorced/separated/widowed).

#### Brief Resilience Scale, BRS

Resilience was the main outcome variable of this study. BRS is a six-item scale measuring an individual’s ability to bounce back after stressful events (39). Items were scored from 1 (Strongly Disagree) to 5 (Strongly Agree). Negatively scored items were reverse scored. Items were summed and averaged, with a higher score indicating a greater level of resilience (mean score range: 1 to 5). A mean score of 1.00 to 2.99 indicates low resilience, 3.00 to 4.30 indicates normal resilience, and 4.31 to 5.00 indicates high resilience, respectively (39). The scale was revealed to have a one-factor structure, strong internal consistency and test–retest reliability (40). This measure has been utilized in various populations in Singapore (41–43). The Cronbach’s alpha was 0.80 for the current study, suggesting strong internal consistency reliability.

#### Patient Health Questionnaire, PHQ-9

PHQ-9 is a nine-item scale, with items scored from 0 to 3 (44). A higher score indicates higher depressive severity. The cumulative scores of all nine items were calculated (score range: 0 to 27). A total score of 10 and above suggests moderate/severe levels of depressive symptoms (45). The PHQ-9 is a valid and reliable screening instrument for depressive symptoms in the general population (46, 47). The Cronbach’s alpha of the scale was 0.83, suggesting strong internal consistency reliability.

#### Generalized Anxiety Disorder Scale, GAD-7

GAD-7 is a seven-item scale, with items scored from 0 to 3 (48). The cumulative scores of all seven items were calculated (score range: 0–21). A higher score indicates higher anxiety severity. A total score of 10 and above suggests moderate/severe levels of anxiety symptoms (49). The scale is a valid and reliable screening tool for anxiety symptoms in the general population (50). The Cronbach’s alpha score for the present sample was 0.87, suggesting strong internal consistency reliability.

#### Depression Anxiety Stress Scale, DASS-21

The DASS-21 comprises 21 items, with seven items for each of the three psychological distress subscales (depression, anxiety, and stress)(51). It has been demonstrated to be a valid screening tool for the general population (52). The present study only utilizes the seven items addressing stress (i.e., DASS-21 stress). The stress scale measures trouble relaxing, tenseness, being easily on edge, irritability, and over-reactivity. Each item is scored from 0 to 3. Items for stress were scored according to the original authors of the scale – the summed numbers in the subscale were multiplied by two before interpreting the scores. The cumulated scores for DASS-21 stress ranges from 0 to 42. Higher scores indicate higher levels of stress. The Cronbach alpha for the stress component was 0.86 in this study, suggesting strong internal consistency reliability.

#### Sources of COVID-19-related stressors

The present study examined four sources of stress that measured whether participants felt anxious due to certain thoughts or concerns stemming from the COVID-19 outbreak (i.e., “In the past month, did you feel anxious due to some of the following thoughts or concerns related to the COVID-19 outbreak?”) (38). Questions included statements relating to common COVID-19-related stressors regarding: (1) The possibility of self/family/friends being infected with COVID-19, (2) The possibility of self/family/friends dying due to COVID-19, (3) Unemployment and/or financial loss, such as losing work opportunities or having to take unpaid leave, (4) School closure. Items were dichotomously scored (no = 0, yes = 1) individually.

### Procedure

**Figure 1:**
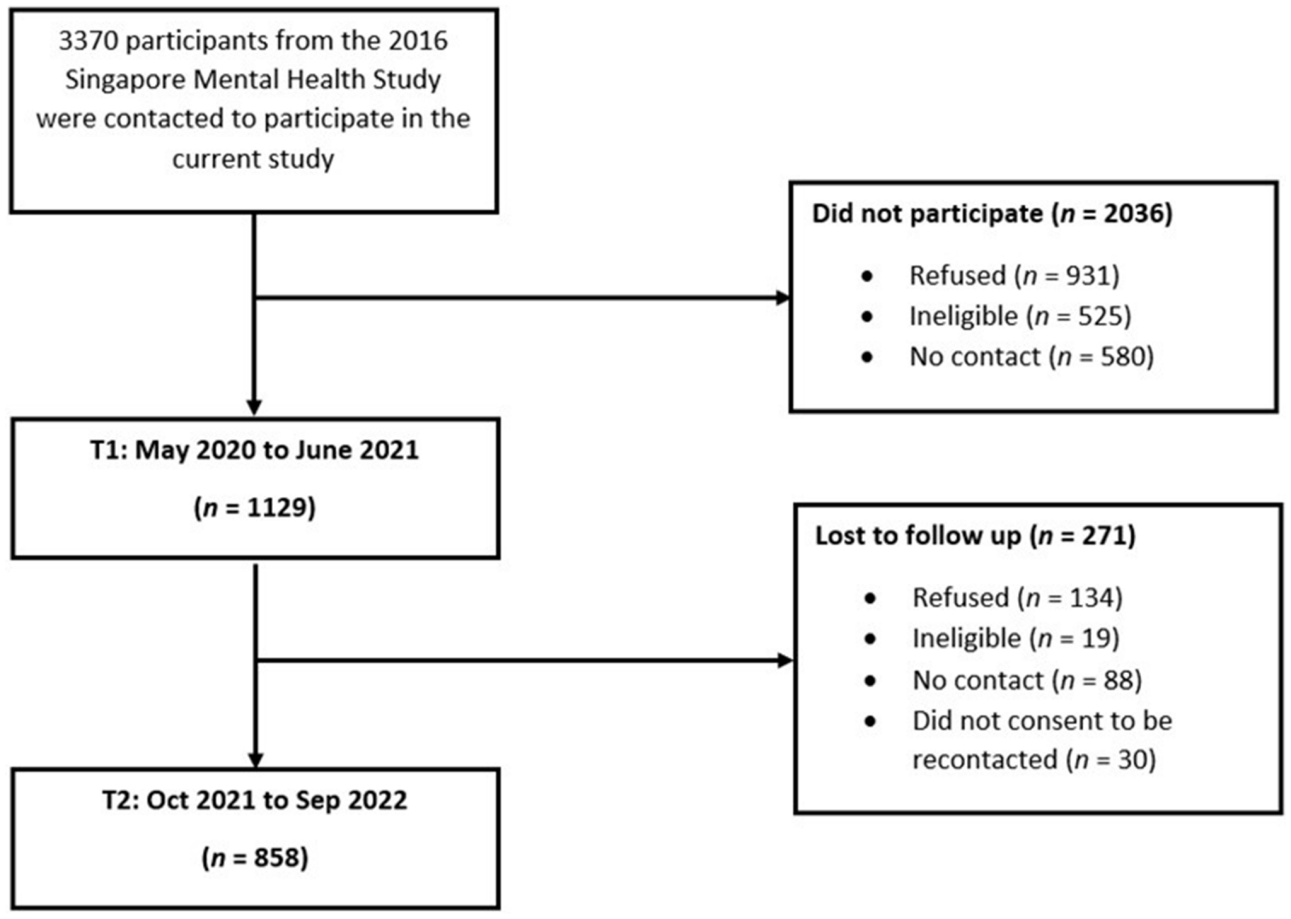
Flowchart of participants’ recruitment to the study.

Participants underwent an interview session for each wave of the study with trained research staff using structured questionnaires either via ZOOM or face-to-face. Most participants opted for ZOOM, but the latter option was provided so as not to exclude participants who may not be technologically savvy, especially older participants. Face-to-face sessions were conducted in the later stages of data collection, once pandemic restrictions permitted it, at locations convenient for each participant (e.g. their homes). Each interview session lasted for about 40–50 minutes. During T1, participants indicated if they were willing to be contacted for future studies. Participants who agreed were recontacted approximately a year after they first took part in the study. Participants were not recontacted for participation in T2 until at least a year had passed since their participation during T1 (e.g., if a participant underwent the interview session in November 2020 for T1, they were recontacted during November 2021 for T2). The first measurement (T1) was carried out from May 2020 to June 2021 when the first wave of the pandemic was receding, and partial lockdown (i.e., circuit breaker) and other Safe Management Measures on the population were starting to be lifted (53, 54). The second measurement (T2) was carried out from October 2021 to September 2022 after the second wave of the pandemic had receded and about 82 to 93% of the population had completed two doses of the COVID-19 vaccines (53, 54). During both sessions, participants were interviewed on their sociodemographic information, psychological distress, COVID-19 stressors, and resilience, among other variables. All participants provided either electronic or physical written informed consent. A flow chart of the recruitment process is given in Figure 1.

### Data analysis

Descriptive statistics of the baseline (T1) were calculated for all the variables. To account for demographic differences between each survey sample and the underlying population, we applied post-stratification weighting and inverse probability of censoring weighting to the data (55). Categorical variables were presented as weighted percentages and unweighted frequency (refer to Table 1). Continuous variables (PHQ-9, GAD-7, DASS-21 stress, BRS) were presented as weighted mean and standard deviation (SD) (refer to Table 2). Variance Inflation Factor scores were below 5 for all predictors, indicating no multicollinearity concerns. A generalized estimating equation (GEE) was performed to investigate the change in resilience across the two time points (T1 = 0, T2 = 1). In the subsequent GEE model, sociodemographic variables, PHQ-9, GAD-7, DASS-21 stress, and COVID-19-related stressors were included to identify the significant longitudinal correlates of resilience.

**Table 2:** Weighted mean and standard deviations of the study instruments.

	Overall			Timepoint 1		Timepoint 2	
	Mean	SD	*p*-value^#^	Mean	SD	Mean	SD
BRS (range 0–5)	3.61	0.62	0.180	3.61	0.63	3.61	0.60
PHQ-9 (range 0–27)	3.17	3.88	0.485	3.07	3.77	3.29	4.01
GAD-7 (range 0–21)	3.24	4.04	0.378	3.18	4.00	3.32	4.09
DASS-21 stress (range 0–42)	5.10	6.36	0.368	5.00	6.17	5.22	6.60

^#p-values were derived from GEE model of the variables and timepoints.^

A sub-analysis was conducted to further investigate the relationship between self-reported symptoms of anxiety and depression and resilience. For this analysis, BRS was categorized into low, normal, and high levels of resilience using the respective cut-off scores, and PHQ-9 and GAD-7 were classified into two categories (moderate/severe and no/mild range). GEE was conducted with BRS categories as a predictor for PHQ-9 and GAD-7, while adjusting for sociodemographic variables and timepoints. Data processing and cleaning were conducted via SAS Version 9.4 for both baseline and follow-up datasets to ensure that the two variable lists were identical for merging. Survey weights were also created using SAS Version 9.4. Thereafter, survey weighted analyses such as tabulate and the mean were performed in STATA S/E Version 15 to populate the numbers for sociodemographic variables and study instruments (Tables 1–3). STATA was also used for the GEE models to investigate associations between resilience and sociodemographic variables (Table 4). Next, using STATA, we predicted for PHQ-9 and GAD-7 cut-offs and resilience categories (Table 5), while adjusting for the following sociodemographic variables in the GEE model: age, gender, ethnicity, educational level, marital status, children, employment status, monthly personal income and housing type. The statistical significance of all analyses was determined at 0.05 level (*p*-value <0.05) using two-sided tests.

**Table 3:** Proportion of participants who had moderate/severe scores for PHQ-9 and GAD-7.

	Overall		Timepoint 1		Timepoint 2	
	%	*n*	%	*n*	%	*n*
GAD-7						
No/Mild (<10)	91.70	1781	91.63	1,019	91.80	762
Moderate/Severe (≥10)	8.29	198	8.37	106	8.19	92
PHQ-9						
No/Mild (<10)	91.99	1798	92.40	1,026	91.45	772
Moderate/Severe (≥10)	8.01	179	7.59	96	8.55	83

**Table 4:** Results from generalized estimating equation model to investigate the association between sociodemographic variables and BRS (*n* = 1,112).

	β coefficient	95% CI		*p*-value
Age groups				
21–34 [ref]				
35–49	−0.04	−0.14	0.07	0.48
50–64	−0.06	−0.18	0.07	0.37
65+	−0.17	−0.33	−0.01	0.045
Gender				
Female [ref]				
Male	0.13	0.04	0.22	0.00
Ethnicity				
Chinese [ref]				
Malay	0.06	−0.05	0.16	0.31
Indian	0.00	−0.08	0.09	0.97
Others	0.00	−0.11	0.11	0.98
Highest education attained				
University and above [ref]				
Below primary school	−0.15	−0.35	0.05	0.14
Secondary school	−0.03	−0.18	0.13	0.73
Pre-University^1^	−0.06	−0.16	0.04	0.25
Marital status				
Never married [ref]				
Married/Cohabiting	−0.05	−0.19	0.09	0.47
Divorced/Widowed/Separated	−0.04	−0.26	0.18	0.72
Do you have any children?				
No [ref]				
Yes	0.11	−0.03	0.26	0.13
Current employment status				
Employed/Self-Employed [ref]				
Unemployed	−0.08	−0.25	0.10	0.39
Economically inactive				
Monthly personal income (SGD)				
2,000 to 3,999 [ref]				
Below 2,000	0.07	−0.04	0.19	0.21
4,000 to 5,999	−0.09	−0.20	0.01	0.09
6,000 to 9,999	−0.07	−0.19	0.05	0.26
10,000 and above	0.05	−0.11	0.21	0.53
Housing type				
HDB^2^ 4/5 room/Executive/Jumbo [ref]				
HDB^2^ 1/2/3 room	0.07	−0.04	0.19	0.21
Private housing	0.11	0.01	0.21	0.03
Psychological variables				
PHQ9^#^	−0.02	−0.04	−0.01	0.00
GAD7^#^	−0.02	−0.03	0.00	0.01
DASS-21 stress^#^	−0.02	−0.03	−0.01	0.00
I/family/friends might be infected with COVID-19 (Yes)^#^	−0.05	−0.12	0.02	0.15
I/family/friends might die due to COVID**-**19 (Yes)^#^	−0.01	−0.09	0.06	0.72
Unemployment/Financial loss, such as losing work opportunities or having to take unpaid leave (Yes)^#^	−0.11	−0.19	−0.02	0.01
School closure (Yes)^#^	0.02	−0.07	0.11	0.70

^1^Pre-university includes Junior College, Vocational Institutes, Institute of Technical Education, and any form of Diploma. ^2^Housing Development Board (HDB) refers to public housing in Singapore. As of 2021, about 80% of Singapore’s population live in houses developed by HDB. ^#^Adjusted for sociodemographic variables. Bold values indicate a statistically significant *p*-value (< 0.05).

**Table 5:** Generalized estimating equations (GEE) model with BRS categories as a predictor for meeting clinical criteria of PHQ-9 and GAD-7.

	PHQ-9 (≥10)					GAD-7 (≥10)				
	Crude β	Adjusted β^#^	95% CI		*p*-value	Crude β	Adjusted β^#^	95% CI		*p*-value
BRS scores										
Normal (range: 3.00-4.30) [ref]										
Low (range: 0–2.99)	1.44	1.43	1.06	1.79	**<0.001**	1.61	1.63	1.28	1.98	**<0.001**
High (range: 4.31–5.00)	−1.32	−1.25	−2.22	−0.27	**0.012**	−1.11	−1.08	−1.94	−0.022	**0.013**

^#^Adjusted for sociodemographic variables, PHQ-9 ≥ 10 = moderate/severe levels of depressive symptoms, GAD-7 ≥ 10 = moderate/severe levels of depressive symptoms. Bold values indicate a statistically significant *p*-value (<0.05).

## Results

During T1, the weighted prevalence of normal levels of resilience was 74.5%. The weighted prevalence of low and high levels of resilience was 13.3% and 12.2%, respectively. During T2, the weighted prevalence of normal levels of resilience was 76.9%. The weighted prevalence of low and high levels of resilience was 12.8% and 10.3%, respectively. The weighted means (SD) of BRS were 3.61 (0.63) for T1 and 3.61 (0.60) for T2. Mean BRS scores did not differ across timepoints. The other psychological measures (i.e., PHQ-9, GAD-7 and DASS-21 stress) also did not differ much across the two timepoints, *p* > 0.05 (Table 2). Overall, 8.3% (*N* = 198) of participants reported moderate/severe anxiety symptoms on the GAD-7, while 8.0% (*N* = 179) reported moderate/severe depressive symptoms on the PHQ-9. Refer to Table 3 for the breakdown of participants in the different score ranges across timepoints.

The GEE analysis revealed that age, gender, and housing type were associated with resilience across the two waves. Older adults (65 years old and above) had lower levels of resilience as compared to their younger counterparts (*β* = −0.17, 95% CI: −0.33 to −0.01). Males had a higher level of resilience compared to females (*β* = 0.12, 95% CI: 0.03 to 0.21). Individuals who reported staying in private housing had higher levels of resilience as compared to individuals staying in public housing (*β* = 0.11, 95% CI: 0.01 to 0.21). Ethnicity, educational level, marital status, having children current employment status, and monthly personal income were not significantly associated with resilience scores. Table 4 reflects the associations between sociodemographic variables and BRS for the present sample while controlling for timepoints.

Secondly, symptoms of anxiety and depression, stress levels, and a specific stressor about COVID-19 were significantly associated with resilience. Higher scores in PHQ-9 (*β* = −0.02, 95% CI: −0.04 to −0.01), GAD-7 (*β* = −0.02, 95% CI: −0.03 to 0.00) and DASS-21 stress (*β* = −0.02, 95% CI: −0.03 to −0.01) were associated with lower scores in BRS. Individuals who were worried about unemployment/financial loss, such as losing work opportunities or having to take unpaid leave (*β* = −0.10 95% CI: −0.20 to −0.01) had significantly lower scores of resilience (see Table 4).

As compared to participants with normal BRS scores (range: 3.00–4.30), participants with low BRS scores (range: 0–2.99) were 1.43 times more likely to report moderate/severe levels of depressive symptoms (*p* < 0.001) and 1.63 times more likely to report moderate/severe levels of anxiety symptoms (*p* < 0.001). Participants with high BRS scores (range: 4.31–5.00) were 1.25 times less likely to report moderate/severe levels of depressive symptoms (*p* = 0.012) and 1.08 times less likely to report moderate/severe levels of anxiety symptoms (*p* = 0.013) as compared to participants with normal BRS scores. The results are reported in Table 5.

## Discussion

The present study investigated the population’s ability to “bounce back” from the multifaceted stressors introduced by the COVID-19 pandemic. With the continued uncertainty in Singapore, coupled with the persistence of COVID-19 in the nation almost two years after its introduction, it would be reasonable to expect a dip in the resilience levels. However, resilience scores were observed to be similar throughout the two years of the pandemic, owing to the population’s ability to recover from the continued and multifaceted stressors brought upon by the pandemic. A longitudinal study conducted in Australia yielded similar results and attributed the consistency in resilience to the ability of the sample to cope and adjust well to the psychosocial and economic impacts of the pandemic (30). Furthermore, a systematic review conducted in 2023 examining the trajectory of anxiety, depression and general mental health symptoms revealed that symptoms remained consistent pre- and post-pandemic across many countries, attributing it to high population resilience (56). In regard to Singapore, the government has been proactive in taking steps to preserve the population’s mental well-being. Substantial efforts were focused on providing resources, raising awareness of mental health, and encouraging help-seeking behaviors such as implementing a mental health resource hub, national crisis hotlines and a ‘COVID-19 Mental Wellness Taskforce’ (57). As such, these initiatives may have contributed to minimizing the impact of the pandemic to an extent, allowing the population to cope and adapt better.

While it is a positive finding that most of the population reported having normal levels of resilience, we cannot ignore vulnerable individuals who are susceptible to poorer resilience. Individuals staying in public housing as compared to private housing reported having lower levels of resilience. Housing type is often used as a proxy for family socioeconomic levels due to its relationship with income status in the nation (58). Interestingly, interconnected socioeconomic factors such as employment, education levels and income were not associated with resilience in the present study. Some possible explanations for such findings could be that, firstly, it was reported that by April 2022, during the second wave of the study, Singapore’s unemployment rates bounced back to pre-pandemic levels (59), possibly suggesting that unemployment was not a contributing factor that affected most residents. The current study also had a small sample of unemployed individuals, further corroborating this. Additionally, in its earlier stages, initiatives to mitigate the economic consequences of the pandemic were introduced in the nation with the hopes of addressing issues such as employment and financial concerns. This included jobs and skills packages, monetary support grants, and COVID-19 recovery grants, especially for lower-income groups (57). These could have alleviated some of the economic consequences of the pandemic, serving as a possible explanation as to why income levels were not associated with resilience levels in the current population. Additionally, a pre-pandemic study revealed that while socioeconomic status can play a role in psychological resilience, it is more closely associated with individual characteristics and adaptive strategies (60). Nonetheless, the disparity in resilience levels among individuals in different housing groups displays some evidence that individuals with lower socioeconomic status displayed lesser resilience.

Additionally, the results revealed that females had lower resilience compared to their male counterparts, consistent with other studies conducted during the pandemic (16, 26, 61). During the pandemic, women reported having more worries, were more susceptible to stressful situations, and elicited poorer implicit and explicit anxiety (13, 62). Women are also more likely to be frontline health workers (e.g., nurses, midwives) and essential health facility workers (e.g., cleaners) (63). Additionally, females feel more pressured to take up the unpaid labor of ensuring the emotional well-being of children, parents, and other family members (64), and mothers were more likely to take on the responsibility of childcare and home education during the closure of childcare centers and schools, adding new pressures for them (64, 65).

It was also revealed that those aged 65 and above had lower resilience scores. A similar nationwide study conducted in China revealed greater psychological distress among older adults during the pandemic (66). Specific to Singapore, another nationwide study also revealed that older adults (aged 56–78) reported having lower levels of social resilience compared to their counterparts aged 26–35 and 46–55 (67). Older adults are typically more vulnerable to the COVID-19 virus, especially those with comorbid illnesses, which further serves as a stressor (68). Furthermore, suspending community and care services and reduced face-to-face interactions with family and friends during the pandemic might have led to increased loneliness among this group who might have relied on these factors to meet their social needs (68).

In general, pandemic-related stressors negatively impacts mental health and distinguishing the specific stressors that have a greater impact on mental health would aid in the development of targeted interventions (69). Findings from the study revealed that individuals who had employment concerns (i.e., worries about unemployment/financial losses such as losing work opportunities or having to take unpaid leave) tended to have lower levels of resilience. This could be interlinked with worries about the rising cost of living reported in 2022 (70). Despite unemployment rates being relatively low in the nation, the unprecedented increase in layoffs seen worldwide during the pandemic might have caused individuals to start assessing the security of their employment (71). A study done in 2020 revealed that higher perceived job insecurity can lead to greater anxiety symptoms because of the intensified worry about one’s financial situation, causing significant psychological distress (72). Lastly, individuals who scored higher on the GAD-7, PHQ-9 and DASS-21 stress measures also had lower levels of resilience. Cross-sectional and longitudinal studies during the pandemic have also yielded similar results, with higher stress, depression and anxiety levels being associated with lower levels of resilience (13, 30, 31). Psychological distress influences one’s ability to cope with situations, especially during adverse events like the pandemic, and thus, these findings were not surprising (30, 73). On top of that, individuals with low resilience were more likely to have moderate to severe levels of depressive and anxiety symptoms, highlighting resilience as a potential protective factor. The capacity of resilience to prevent psychopathology and maintain mental well-being has been previously documented (74). Other similar studies have also revealed that subgroups of individuals with depression and anxiety have lower levels of resilience (75, 76). This highlights the need for nurturing resilience in mitigating depressive and anxiety symptoms. Conceptualizing resilience as “the ability to bounce back” from adverse circumstances can be beneficial in guiding the development of interventions that aim to enhance resilience when it is viewed as an ability (77). The normal levels of resilience among the adult population that remained mostly unchanged throughout the pandemic are somewhat indicative that efforts in promoting mental well-being in the nation have been beneficial. During the partial lockdown period, downstream initiatives were developed to target specific groups, such as those at risk of developing or those with pre-existing mental health conditions. Increased funding was directed to mental healthcare, and various agencies were set up to triage and deliver initial interventions (57). There was also an increase in helplines to provide support for those with anxiety, mood, and other mental health-related struggles (57). A meta-analysis conducted in 2020 revealed that approximately one-third of the global population experienced stress, anxiety and/or depression as a result of the pandemic (78, 79). The prevalence of psychological distress, coupled with its influence on resilience, highlights a need for continuous and enhanced health promotion involving varying community and primary mental health services that screen for and target people who are at risk.

Additionally, nationwide initiatives included financial and employment support, rolled out during the early stages of the pandemic. This included jobs and skills packages, monetary support grants, and COVID-19 recovery grants (57). Providing financial support to prevent deprivation allows individuals to adopt protective behaviors and better cope with changing environments, which are salient for resilience (80). More policies and initiatives can be rolled out in support of affected individuals to help them better cope and build their resilience. Lower resilience among females and older adults also calls for more targeted gender-sensitive measures and more promotion of late-life coping during adversities like these to provide social, psychological and economic support for these groups. For example, following the COVID-19 pandemic, there has been an emphasis that public health response strategies should be inclusive of women’s health and address issues such as gender norms and the need for shared responsibilities at home and work, and prioritizing female frontline healthcare workers’ mental health (81, 82). Additionally, it has been suggested that accessible telehealth interventions can also be implemented to mitigate social loneliness and increase psychological resilience among older adults (80, 83).

There are some limitations to this study. Firstly, the self-reported measures are limited by their biases. Secondly, the first survey commenced in May 2020, at least 3 months after the first case of COVID-19 was reported in the nation and approximately 2 months after the World Health Organization declared COVID-19 a pandemic (1, 84). It is not unlikely that resilience levels changed from the beginning of the pandemic till T1. Notwithstanding these, the study has several strengths. This is one of the first studies in Singapore to explore psychological resilience among its adult population during the pandemic. The study collected within-individual data across months after the outbreak, which sheds some light on the trajectory of resilience and the short-term effects of the policies and steps that were put in place to mitigate the mental health impact of the pandemic. Additionally, we captured data from a large nationally representative sample, utilizing valid and reliable measures of resilience and psychological distress.

The present study gives us a better picture of the short-term impact of the pandemic on individual resilience among the Singapore adult population. The study revealed that psychological resilience remained the same 1 year after lockdown measures were lifted, with most of the population depicting normal levels of resilience. Several sociodemographic and psychological variables were influential in promoting better resilience. These findings reflect the factors that need to be targeted to better allow individuals to cope with not only the COVID-19 pandemic but adversities of a similar nature. More empirical research is needed over the next few years to understand the long-term psychological effects of the pandemic. Continued efforts to address resilience are essential in understanding and improving individual coping and well-being in a post-pandemic world.

## Data Availability

The datasets presented in this article are not readily available because the authors’ government law and institution only permits the sharing of human participant data with researchers with whom they have a Research Collaboration Agreement (RCA). However, data sharing with clear research purposes can be made available upon request. Requests to access the datasets should be directed to mythily@imh.com.sg.
